# Real-World Efficacy and Safety of Thoracic Radiotherapy after First-Line Chemo-Immunotherapy in Extensive-Stage Small-Cell Lung Cancer

**DOI:** 10.3390/jcm12113828

**Published:** 2023-06-02

**Authors:** Zhaoliang Xie, Jingru Liu, Min Wu, Xiaohan Wang, Yuhan Lu, Chunyan Han, Lei Cong, Jisheng Li, Xue Meng

**Affiliations:** 1 Department of Radiation Oncology, Shandong Cancer Hospital and Institute, Shandong First Medical University and Shandong Academy of Medical Science, Jinan 250117, China; 2 Department of Radiation Oncology, Shandong Cancer Hospital and Institute, Shandong University Cancer Center, Jinan 250117, China; 3 Suzhou Cancer Center Core Laboratory, The Affiliated Suzhou Hospital of Nanjing Medical University, Suzhou 215000, China; 4 Department of Emergency Medicine, The First People’s Hospital of Neijiang, Neijiang 641099, China; 5 Department of Radiotherapy, The Third Affiliated Hospital of Shandong First Medical University, Jinan 250031, China; 6 Department of Oncology, Shandong Provincial Hospital Affiliated to Shandong First Medical University, Jinan 250117, China; 7 Department of Medical Oncology, Qilu Hospital of Shandong University, Jinan 250012, China

**Keywords:** extensive-stage small-cell lung cancer (ES-SCLC), thoracic radiotherapy (TRT), chemo-immunotherapy (CT-IT), survival, adverse event

## Abstract

(1) Background: At present, the efficacy and safety of thoracic radiotherapy (TRT) after chemo-immunotherapy (CT-IT) in patients with extensive-stage small-cell lung cancer (ES-SCLC) still remain unclear. The purpose of this study was to evaluate the role of TRT after CT-IT in patients with ES-SCLC. (2) Methods: From January 2020 to October 2021, patients with ES-SCLC treated with first-line anti-PD-L1 antibody plus platinum-etoposide chemotherapy were enrolled retrospectively. The survival data and adverse events data of patients treated with or without TRT after CT-IT were collected for analysis. (3) Results: A total of 118 patients with ES-SCLC treated with first-line CT-IT were retrospectively enrolled, with 45 patients with TRT and 73 patients without TRT after CT-IT. The median PFS and OS in the CT-IT + TRT group and CT-IT only group were 8.0 months versus 5.9 months (HR = 0.64, *p* = 0.025) and 22.7 months versus 14.7 months (HR = 0.52, *p* = 0.015), respectively. The median PFS and OS in all 118 patients treated with first-line CT-IT were 7.2 and 19.8 months with an ORR of 72.0%. In multivariate analyses, liver metastasis and response to CT-IT were shown to be independent prognostic factors of PFS (*p* < 0.05), while liver metastasis and bone metastasis were independent predictive factors of OS (*p* < 0.05). Although TRT was significantly associated with better PFS and OS in univariate analysis, the association of TRT and OS failed to reach statistical significance (HR = 0.564, *p* = 0.052) in multivariate analysis. There was no significant difference in adverse events (AEs) between two treatment groups (*p* = 0.58). (4) Conclusions: ES-SCLC patients treated with TRT after first-line CT-IT had prolonged PFS and OS with an acceptable safety profile. Further prospective randomized studies are necessary to explore the efficacy and safety of this treatment modality for ES-SCLC in future.

## 1. Introduction

Small-cell lung cancer (SCLC) accounts for 13–17% of all lung cancer with the majority of patients diagnosed at an extensive disease stage [1,2]. It is characterized by early tumorous spread and rapid cell multiplication. Only 16% of patients with SCLC survive more than 5 years after diagnosis [3]. The standard first-line treatment is platinum chemotherapy (carboplatin or cisplatin) with etoposide, but limited progress has been made in more than three decades [4]. At present, immune checkpoint inhibitors have become a new therapeutic method for ES-SCLC. In IMpower133 and CASPIAN study, it has been confirmed that anti-programmed cell death ligand-1 (PD-L1) antibody atezolizumab or durvalumab combined with platinum-etoposide chemotherapy significantly prolonged both PFS and OS and reduced the risk of disease progression and death without significantly increasing the incidence of AEs as first-line treatment of ES-SCLC [5,6]. Therefore, atezolizumab or durvalumab plus chemotherapy has already been approved for first-line treatment of ES-SCLC [7]. However, the overall survival of ES-SCLC with chemo-immunotherapy (CT-IT) has remained unsatisfying. The disease of half of the patients receiving first-line CT-IT would progress within 6 months and the median overall survival of first-line CT-IT was only between 12 and 13 months [8]. Thus, there is a huge unmet need for novel treatment modality of ES-SCLC.

TRT plays an important role in the comprehensive treatment of ES-SCLC. In recent studies, it has been shown that TRT was beneficial to locoregional control and survival in patients who have completed first-line platinum-based chemotherapy [9]. For example, the CREST trial shows that TRT significantly improved the locoregional control and the survival of patients who responded to chemotherapy [10]. In this study, 87% of ES-SCLC patients had persistent intrathoracic disease left after chemotherapy. A non-randomized phase 2 clinical trial and a retrospective analysis also show that TRT is beneficial and safe for patients with ES-SCLC [11,12]. Evidence-based guidelines for SCLC treatment recommend that consolidated TRT could be given to patients who responded to initial chemotherapy [13].

However, the efficacy and safety of TRT following chemo-immunotherapy induction still remain unknown, since TRT was not allowed during immunotherapy maintenance in both IMpower133 and CASPIAN study design [5,6]. In the immunotherapy era of ES-SCLC, by now there have been seldom studies evaluating the efficacy and safety of thoracic radiotherapy after first-line chemo-immunotherapy induction in ES-SCLC patients. Herein, we performed a retrospective study to evaluate the efficacy and safety of TRT in ES-SCLC patients following first-line CT-IT in a real-world setting.

## 2. Methods

### 2.1. Patients

Medical records of patients who were diagnosed with ES-SCLC and treated with chemotherapy and anti-PD-L1 antibodies at three hospitals in Shandong Province of China (Shandong Cancer Hospital, Qilu Hospital of Shandong University, and The Third Affiliated Hospital of Shandong First Medical University) from January 2020 to October 2021 were included in this retrospective multicenter study. Brain magnetic resonance imaging (MRI), contrast-enhanced thoracic and abdominal computed tomography (CT), and bone radionuclide imaging (ECT) or positron emission tomography (PET)/CT were used to assess the initial staging of the patients and the treatment efficacy. The inclusion criteria were as follows: (1) patients were aged 18 years or older with histologically or cytologically confirmed SCLC; (2) ES-SCLC confirmed based on the Veterans Administration Lung Study Group [VALG] staging system; (3) patients who achieved partial response (PR), complete response (CR), stable disease (SD), or progressive disease (PD) after platinum-based CT-IT were included. The exclusion criteria were as follows: (1) a history of malignancy in other sites (before or at the same time); (2) not a single histological type; (3) receiving CT-IT other than the first-line setting; (4) receiving immune checkpoint inhibitor treatment other than anti-PD-L1 antibody. Eligible patients were divided into two groups: CT-IT plus TRT group, which included patients who received TRT after receiving CT-IT, and CT-IT group, which included a control group of patients who received CT-IT alone. The study was approved by the Ethics Committee of Shandong Cancer Hospital and was conducted in accordance with the Declaration of Helsinki.

### 2.2. Treatment

All patients were treated with durvalumab, atezolizumab, or other anti-PD-L1 antibody plus platinum-etoposide chemotherapy followed by maintenance of anti-PD-L1 antibody until the occurrence of disease progression according to Response Evaluation Criteria for Solid Tumors (RECIST) version 1.1 criteria or unacceptable toxic events [14]. The doses and adjustments of regimens was decided by doctor’s choice based on the guidelines of the National Comprehensive Cancer Network or the Chinese Society of Clinical Oncology. In regard to radiation techniques, intensity-modulated radiotherapy (IMRT) was used for TRT. The organs at risk (OARs) and target volumes were defined based on the Radiation Therapy and Oncology Group guidelines for lung cancer. The residual primary tumor after CT-IT and the positive lymph nodes were included in the gross target volume (GTV). The clinical target volume (CTV) was expanded from GTV with a 0.5–0.8 cm margin and included all the positive regional nodal regions at diagnosis. The planning target volume (PTV) was increased by 0.5–1 cm from the CTV. The radiation therapy plans were verified to ensure adequate coverage of the prescribed radiation dose was at least ninety-five percent of the PTV. The total radiation dose ranges from 30 Gy to 60 Gy, with a median dose of 45 Gy, at 2 Gy to 3 Gy per fraction once daily (five fractions per week). The radiation dose of OARs was constrained as follows: maximal radiation dose of the spinal cord was ≤45 Gy, the mean radiation dose of the lung was <17 Gy, V5 to total lungs ≤ 60%, V20 to total lungs ≤ 30%, V30 to total lungs ≤ 20%, mean radiation dose of heart was ≤20 Gy, V30 to heart ≤ 40%, V40 to heart ≤ 30%, mean radiation dose of the esophagus was ≤30 Gy, V60 to esophagus ≤ 17%.

### 2.3. Information Collection and Follow-Up

Patients’ clinical characteristics were recorded including age, sex, smoking, alcohol usage, height, weight, body mass index (BMI), tumor-node-metastasis (TNM) stages, ECOG scores and metastatic site, white blood cell (including neutrophil, lymphocyte, monocyte) counts, and platelet counts, as well as hemoglobin and lactate dehydrogenase (LDH) level, cycles of CT-IT, response to CT-IT and TRT. The evaluation of clinical response to treatment was performed according to the Response Evaluation Criteria in Solid Tumors (RECIST) 1.1 criteria. PFS was measured from the first date of receiving CT-IT to the date of progressive disease, death, or the final follow-up endpoint, whichever occurred first. OS was measured from the first date of receiving CT-IT to the date of death, loss to follow-up, or the final follow-up endpoint, whichever occurred first.

### 2.4. Statistical Analysis

The data of patients’ baseline characteristics were evaluated by Pearson’s chi-square test for categorical variables or Student’s *t*-test for continuous variables. The PFS curve and the OS curve were described by the Kaplan–Meier method. The log-rank test was used to assess differences between curves. The cut-off values of hematologic markers were determined by the receiver operating characteristic (ROC) curves. Multivariate analysis and univariate analysis were performed to assess the relationship between the clinicopathologic characteristics of patients (including hematologic markers) and survival outcomes using the Cox regression model. Treatment-related toxicity was assessed based on National Cancer Institute Common Terminology Criteria for Adverse Events, version 5.0. A *p*-value of less than 0.05 was defined as statistically significant. All statistical analyses were performed by SPSS Statistics version 25.0 (SPSS Inc., Chicago, IL, USA).

## 3. Results

### 3.1. Patient Characteristics

**Figure 1:**
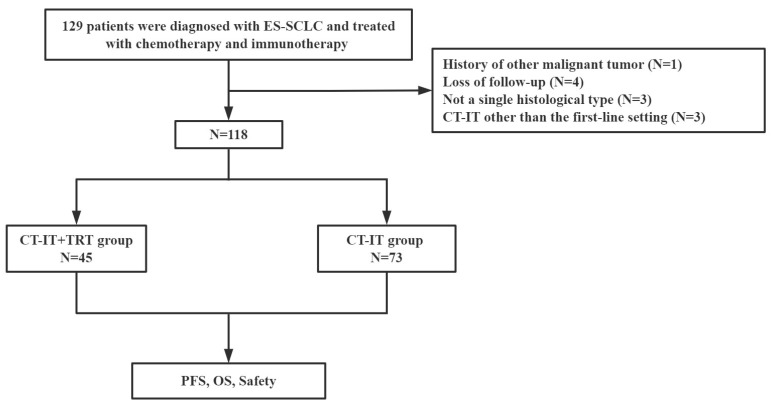
The flow chart of patient selection. Abbreviations: ES-SCLC: extensive-stage small-cell lung cancer; PFS: progression-free survival; OS: overall survival; TRT: thoracic radiotherapy; CT-IT: chemo-immunotherapy.

The information of a total of 129 patients was initially recorded for analysis in the study, but 11 patients were excluded, as shown in Figure 1. Finally, 118 patients were enrolled in our study with 45 patients in the CT-IT + TRT group and 73 patients in the CT-IT group (Figure 1). The baseline clinicopathologic characteristics of all patients are summarized in Table 1. The median age of all patients was 62 years old with 34.7% of patients above 65 years and 81.3% of male patients. Among all patients, 83.0% had a smoking history. The majority of patients (79.7%) had an Eastern Cooperative Oncology Group Performance Status (ECOG PS) ≤ 1. Lung, bone, brain, liver, distant lymph node (LN), and extrathoracic metastases were present in 13.5%, 29.6%, 28.8%, 28.8%, 26.3%, and 89.0% of patients, respectively. A total of 114 (96.6%) patients received four to six cycles of CT-IT. Ninety-one (77.0%) patients received durvalumab or atezolizumab immunotherapy. The CR/PR and SD/PD rates after the initial CT-IT were 72.0% and 28.0%, respectively. Patients in the CT-IT plus TRT group had better ORR (CR + PR) of 84.4% compared with CT-IT only group. In patients with SD/PD response after CT-IT, only 7 patients (all SD) received TRT treatment. Twelve patients in the CI-IT plus TRT group underwent concurrent immunotherapy and TRT. Immunotherapy was stopped during TRT for 33 patients. After TRT, immunotherapy was continued for 15 patients with a median interval of 34 days. Fourteen patients did not receive immunotherapy after TRT due to disease progression, while immunotherapy was discontinued for two patients after TRT due to pneumonia and for another two patients because of death.

### 3.2. Progression-Free Survival Analysis

**Figure 2:**
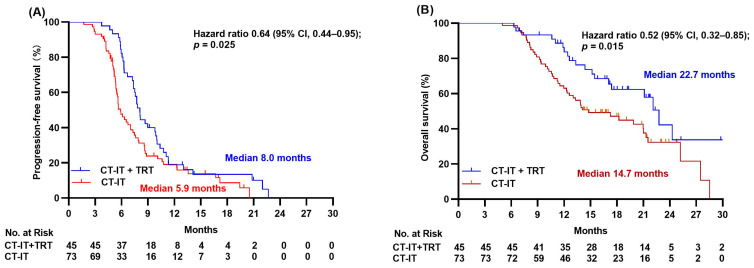
Kaplan–Meier survival curves of progression-free survival (**A**) and overall survival (**B**) of ES-SCLC patients in two treatment groups.

**Figure 3:**
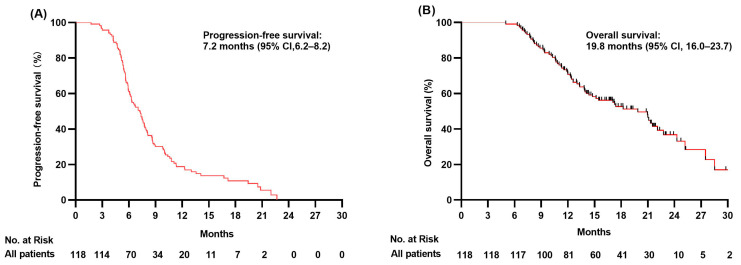
Kaplan–Meier survival curves of progression-free survival (**A**) and overall survival (**B**) for all 118 enrolled patients with ES-SCLC.

The median follow-up time was 21.0 months at data cut-off date of 20 October 2022. Median PFS was 8.0 months in CT-IT + TRT group versus 5.9 months in CT-IT group respectively (HR = 0.64, 95% confidence interval CI 0.44–0.95, *p =* 0.025; Figure 2A). The 6-month and 12-month PFS rates in the two groups were 82.2% versus 45.2% and 17.7% versus 16.4%. Median PFS was 7.2 months for all 118 ES-SCLC patients enrolled in this study (95% CI 6.2–8.2; Figure 3A). Univariate survival analysis was used to determine the association between PFS and clinical features including gender, age, smoking and drinking status, T stage, N stage, BMI, lung metastasis, extrathoracic metastasis, bone metastasis, brain metastasis, liver metastasis, distant LN metastasis, TRT, response to CT-IT, NLR, MLR, PLR, and LDH. Liver metastasis, TRT, and response to CT-IT were found to be significant prognostic factors for PFS (all *p* < 0.05, Table 2). However, multivariate analysis using these three characteristics as parameters revealed that liver metastasis status and response to CT-IT but not TRT were independent prognostic factors of PFS, as shown in Table 2.

### 3.3. Overall Survival Analysis

With a median follow-up time of 21.0 months, the median OS was 22.7 months in CT-IT + TRT group versus 14.7 months in CT-IT group, respectively (HR = 0.52, 95% CI 0.32–0.85, *p* = 0.015; Figure 2B). The OS was 8 months longer in the CT-IT + TRT group than that in the CT-IT group. The 12-month OS rate was 77.7% in the CT-IT + TRT group and 63.0% in the CT-IT group. The median OS was 19.8 months (95% CI 16.0–23.7; Figure 3B) for all 118 ES-SCLC patients enrolled in this study. Univariate survival analysis was used to determine the association between OS and clinical features including gender, age, smoking and drinking status, T stage, N stage, BMI, lung metastasis, extrathoracic metastasis, bone metastasis, brain metastasis, liver metastasis, distant LN metastasis, TRT, response to CT-IT, NLR, MLR, PLR, and LDH. N stage, extrathoracic metastasis, bone metastasis, liver metastasis, TRT, and LDH level were all found to be significant prognostic factors for OS (all *p* < 0.05, Table 3). However, multivariate analysis using above characteristics as parameters revealed that only bone metastasis and liver metastasis were statistically significant independent prognostic factors of OS (both *p* < 0.05), as shown in Table 2. Even though TRT was not shown to be statistically significant independent prognostic factor of OS, it is noticed that the association of TRT with OS nearly reached statistical significance (HR = 0.564, *p* = 0.052) in multivariate analysis.

### 3.4. Survival Outcomes in Selected Patient Subgroups

**Figure 4:**
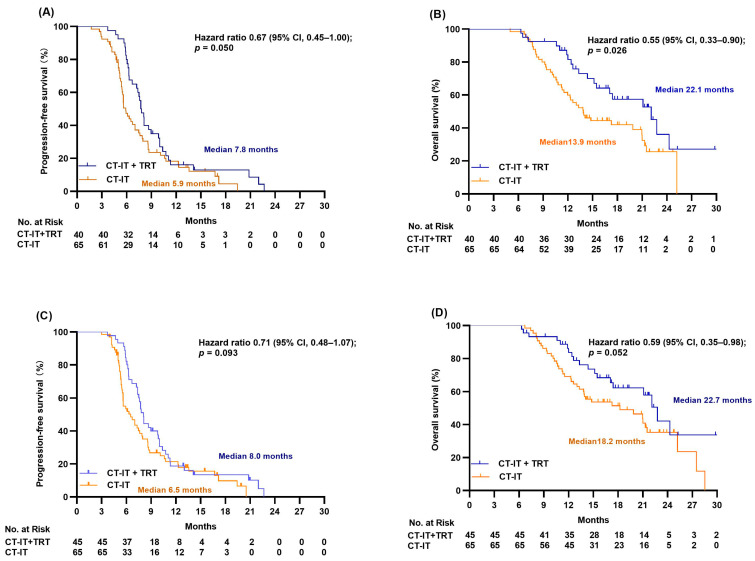
Kaplan–Meier survival curves of progression-free survival (**A**) and overall survival (**B**) in two treatment groups of patients with extrathoracic metastasis. Kaplan–Meier survival curves of progression-free survival (**C**) and overall survival (**D**) in two treatment groups of patients with CR/PR/SD response after CT-IT.

We then analyzed the association between TRT and survival in patients having extrathoracic metastases. For PFS, there was a tendency of PFS improvement in the CT-IT + TRT group (7.8 versus 5.9 months, HR 0.67, *p* = 0.050; Figure 4A). The median OS for patients with extrathoracic metastases was 22.1 months in the CT-IT + TRT group versus 13.9 months in the CT-IT group, respectively (HR 0.55, 95% CI 0.33–0.90, *p* = 0.026; Figure 4B). The association between TRT and survival in patients with CR/PR/SD response after CT-IT was also examined. PFS (8.0 versus 6.5 months, HR 0.71, *p* = 0.093; Figure 4C) and OS (22.7 versus 18.2 months, HR 0.59, *p* = 0.052; Figure 4D) both exhibited a tendency to improve in the CT-IT plus TRT group for patients whose disease did not progress upon first-line CT-IT.

### 3.5. Safety

Adverse events of grades 3 or 4 occurred in 17 (37.7%) of 45 patients in the CT-IT + TRT group and 24 (32.8%) of 73 patients in the CT-IT group. The difference between the two groups was not statistically significant (*p* = 0.58) (Table 4). Hematological toxicities of grade 3 or 4 occurred in 14 (31.1%) patients in the CT-IT + TRT group and 24 (32.8%) patients in the CT-IT group (*p* = 0.84). Pneumonitis and esophagitis were the most common radiation-related toxicities. Two patients developed grade 3 pneumonitis and one patient developed grade 3 esophagitis in the CT-IT + TRT group, while one patient in the CT-IT group developed grade 3 pneumonitis.

## 4. Discussion

As an aggressive tumor with poor prognosis, the median overall survival of first-line etoposide and platinum chemotherapy in ES-SCLC was only 9–11 months [15]. Even when immunotherapy was added to first-line chemotherapy, the median overall survival only reached 12–13 months which is far from satisfying [5,6]. There are currently limited therapeutic choices available for ES-SCLC patients progressed on or after first-line CT-IT. Increasing the efficiency of first-line CT-IT and delaying disease recurrence were essential to improve the prognosis of this deadly malignancy. Previously, the CREST trial showed that TRT after first-line chemotherapy could raise local control rates and improve overall survival [10]. With the arrival of the era of first-line CT-IT for SCLC, a major research interest is whether TRT following CT-IT is effective and safe in ES-SCLC. In fact, there have already been several studies reported which retrospectively investigated this question and some relevant prospective studies are ongoing now.

In the context of first-line chemotherapy, several studies have explored the role of TRT after etoposide and platinum in ES-SCLC. In the CREST trial, eligible patients who responded after four to six cycles of carboplatin or cisplatin with etoposide were randomly assigned to receive either TRT with 30 Gy in 10 fractions or no TRT [10]. Although the primary endpoint of 1-year survival was not significantly improved with the addition of TRT, the 6-month PFS (24% vs. 7%, *p* = 0.001) and 2-year OS (13% vs. 3%, *p* = 0.004) of the TRT group were significantly better compared with the control group, supporting the addition of TRT for patients with good response to chemotherapy. In another trial conducted by Jeremic et al. in 1999, eligible patients who showed a favorable response (CR/PR at the local level and CR at the distant level) after three cycles of chemotherapy were randomly divided into two groups: chemotherapy with EP regimen alone and chemotherapy with EP regimen along with consolidation TRT (54 Gy/36 fractions) [16]. Median OS was significantly improved in the TRT group compared with the observation group (17 vs. 11 months, *p* = 0.041), and the 5-year survival rate was also improved in the TRT group (9.1% vs. 3.7%, *p* = 0.41). Local control was better in the TRT group, but the difference narrowly missed statistical significance (20% vs. 8.1%, *p* = 0.06). However, it is important to be aware that in this study, approximately 90% of patients enrolled had only 1–2 baseline metastases before initial chemotherapy and all patients were treated with prophylactic cranial irradiation (PCI). In the Phase II trial RTOG 0937, ES-SCLC patients with one to four extracranial metastases reaching a complete response or partial response after chemotherapy were randomized to receive TRT (30–45 Gy/10/15f) plus PCI (25 Gy/10f) vs. PCI (25 Gy/10f) alone [17]. Unfortunately, the study’s 1-year OS futility border was crossed at the intended interim analysis, which resulted in the early termination of this study. There were no significant differences between 1-year OS rates of 60.1% for PCI and 50.8% for PCI + TRT (*p* = 0.21), although the PCI + TRT group had an advantage in delaying time to progression.

Although the enrolled population, the dose of radiotherapy, as well as the results differed greatly in these studies, current treatment guidelines worldwide recommend TRT in selected ES-SCLC patients who responded well to first-line chemotherapy, especially those with residual thoracic disease and low-bulk extrathoracic metastatic disease [8,18,19]. However, in the era of first-line chemo-immunotherapy for ES-SCLC, the efficacy and safety of consolidative thoracic radiotherapy still remains largely unknown. There have been few prospective studies investigating the role of consolidative TRT in ES-SCLC patients receiving CT-IT, since TRT was not permitted after CT-IT in pivotal ES-SCLC trials including IMpower133, CASPIAN, ASTRUM005, and CAPSTONE-1 studies [5,6,20,21]. However, there is growing evidence supporting the synergistic effects of immunotherapy in combination with radiotherapy for the treatment of cancer [22,23]. Additionally, some clinical research results have been reported to support the role of this combination modality in lung cancer, particularly non-small cell lung cancer.

It has been shown that radiotherapy could up-regulate the expression of tumor antigens on the surface of tumor cells and strengthen the binding of antibody to tumor cells [22,23]. Meanwhile, immunotherapy could enhance the abscopal effect of radiotherapy [24]. Preclinical data showed the combination of radiotherapy and immunotherapy played a synergistic effect leading to improved tumor control [25,26]. Most current clinical trials demonstrating the benefits of immunotherapy in combination with radiotherapy have focused on non-small cell lung cancer. In the KEYNOTE-001 study, PFS and OS were evaluated according to previous radiotherapy history before immunotherapy [27]. Both PFS and OS were considerably longer for patients with previous radiotherapy than those without radiotherapy (OS: 10.7 vs. 5.3 months, *p* = 0.026; PFS: 4.4 vs. 2.1 months, *p* = 0.019), suggesting that radiation could increase the effectiveness of pembrolizumab in NSCLC patients. In the PEMBRO-RT phase II study, PFS and OS were evaluated in patients treated with pembrolizumab after stereotactic body radiation or pembrolizumab alone [28]. Although there was no statistical significance for PFS or OS between two groups, the ORR at 12 weeks was 36% in the experimental arm vs. 18% in the control arm (*p* = 0.07). The efficacy and safety of durvalumab treatment following chemoradiotherapy in Stage III unresectable NSCLC were assessed in the PACIFIC study, which demonstrated a statistically significant benefit in both PFS and OS for the experimental group receiving durvalumab following chemoradiotherapy [29,30]. Based on the results of this trial, the NCCN guidelines recommend durvalumab treatment following chemoradiotherapy for patients with stage III unresectable NSCLC who did not have disease progression after concurrent chemoradiotherapy [31].

So far, there have been no available randomized controlled clinical study data addressing the efficacy and safety of consolidative TRT in small-cell lung cancer, although some related clinical trials in ES-SCLC are now ongoing [32,33,34]. However, several retrospective studies have been conducted and reported most recently. Daher et al. conducted a multi-center, academic-initiated retrospective study investigating the efficacy and safety of TRT in ES-SCLC [35]. A total of 126 ES-SCLC patients treated with CT-IT were included with 25 patients in the TRT group and 101 patients in the control group. Median PFS was 8.5 months for the TRT group and 5.6 months for the control group (HR 0.48, *p* < 0.003). Median OS was 27.7 months for the TRT group and 13.2 months (HR 0.33, *p* < 0.007) for the control group. Their study showed patients undergoing TRT after CT-IT had a significantly longer PFS and OS with an acceptable safety profile. Bruni et al. also conducted a retrospective study evaluating clinical outcomes and safety of CT-IT with or without TRT in ES-SCLC [36]. Median PFS and OS were 5.4 months and 7.7 months for all the enrolled 31 patients, while the estimated 1-year PFS and OS rate were 37,1% and 33.3%. TRT was shown to be a positive statistically significant prognostic factor for PFS and OS, but the exact PFS and OS time was not informed in this study. Meanwhile, no significant differences in terms of symptomatic side effects were found between patients with or without TRT. In another retrospective study conducted by Wu et al., 22 ES-SCLC patients treated with CT-IT were enrolled, including 11 with and 11 without thoracic radiotherapy. It was revealed that patients with thoracic radiotherapy had a significantly longer median OS (not-reached vs. 9.6 months, *p* < 0.001) than patients without thoracic radiotherapy [37].

In the present retrospective study, we included a total of 118 ES-SCLC patients treated with first-line CT-IT, with 45 patients receiving following TRT, which was much more than the 26 patients receiving CT-IT plus TRT in the study of Daher et al. The median PFS and OS in the CT-IT + TRT group and CT-IT only group of this study were 8.0 months vs. 5.9 months (HR = 0.64, *p* = 0.025) and 22.7 months vs. 14.7 months (HR = 0.52, *p* = 0.015), which were comparable to the results of the study of study of Daher et al. [35]. Although the association of TRT and OS failed to reach statistical significance in multivariate analysis, we detected a strong tendency of significant survival benefit in TRT group (HR = 0.564, *p* = 0.052), which was quite valuable for further investigation in future. For patient selection, liver metastasis and bone metastasis were shown to be independent negative predictive factors of OS in multivariate analysis, suggesting patients with liver and bone metastasis might not benefit from TRT consolidation after CT-IT. In addition, the median PFS and OS in all 118 patients treated with first-line CT-IT in a real-world setting in the present study were 7.2 and 19.8 months with an ORR of 72.0%, which were better compared with first-line chemo-immunotherapy registration RCTs and further supported the role of CT-IT as a standard therapeutic choice for ES-SCLC.

Previous study implicated that immunotherapy in combination with thoracic radiotherapy might increase the risk of pneumonitis [38], thus, the safety of TRT following CT-IT was a crucial and concerned question waiting for more evidence. The safety profile of TRT following CT-IT was generally acceptable and manageable in previously reported retrospective studies mentioned above as well as the present study. For pneumonitis, two instances of grade 3 pneumonitis were observed in the CT-IT plus TRT group in our study, while no pneumonitis cases were reported as related to consolidative TRT in the study of Daher et al. Although there were 6 patients with pneumonitis reported in the retrospective study of Wu et al., most of them were diagnosed with grade 1 and 2 pneumonitis with only one case of grade 4 pneumonitis which was promptly well-managed. The generally acceptable safety and satisfying efficacy in above retrospective studies and our result warranted the necessity of further investigation for the modality of CT-IT plus TRT in ES-SCLC.

There are several limitations with the current study. First, this was a retrospective study with a comparatively small sample size. Thus, the results should be interpreted cautiously and should be further verified in future prospective randomized and controlled study with large sample size. Second, treatment heterogeneity could not be avoided in such a retrospective analysis, such as the first-line CT-IT cycles, other local treatments other than TRT, and later-line medical treatments. Patient outcomes, especially the OS, might be partly impacted by these factors. Third, the total dose and dose per fraction of TRT have varied among patients receiving TRT. These inconsistencies might also make it difficult to draw an exact conclusion for the efficacy and safety of TRT after CT-IT. Fourth, the median follow-up time for this study was comparatively short. A proportion of patients were still alive or the disease had not progressed by the cut-off date. Fifth, limited post-therapy scanning was unavoidable in a retrospective study analyzing data from real-world oncology clinical practice. The timing of the patient’s follow-up imaging examination following treatment or insufficient scans performed could have an influence on the results, especially PFS. Finally, patient selection bias was also one of the important limitations in this study. ES-SCLC patients with better physical conditions and better treatment efficacy after first-line CT-IT were generally more likely to be recommended for TRT after CT-IT.

In conclusion, this retrospective study showed the addition of thoracic radiotherapy following first chemo-immunotherapy was associated with prolonged PFS and OS as well as generally acceptable and manageable safety profile. Although TRT was not significantly associated with improved OS in multivariate analysis, we detected a strong tendency of significant survival benefit for TRT. Future prospective randomized study with large sample size is needed to further explore the efficacy and safety of this potentially promising treatment modality for ES-SCLC.

## Figures and Tables

**Table 1:** Clinicopathological characteristics of all enrolled patients with ES-SCLC.

Characteristics	Total	CT-IT + TRT (%)	CT-IT (%)	*p*-Value
	N = 118	N = 45	N = 73	
Median age (range)	62 (45–90)	62 (45–79)	63 (45–90)	
Age (years)				0.80
>65	41 (34.7)	15 (33.3)	26 (35.6)	
≤65	77 (65.3)	30 (66.7)	47 (64.4)	
Gender				0.43
Male	96 (81.3)	35 (77.8)	61 (83.6)	
Female	22 (18.7)	10 (22.2)	12 (16.4)	
Smoking status				0.02
Smoker	98 (83.0)	33 (73.3)	65 (89.0)	
Never smoker	20 (17.0)	12 (26.7)	8 (11.0)	
Drinking				0.05
Yes	39 (33.1)	10 (22.2)	29 (39.7)	
No	79 (66.9)	35 (77.8)	44 (60.3)	
ECOG PS				0.94
0–1	94 (79.7)	36 (80.0)	58 (79.5)	
2	24 (20.3)	9 (20.0)	15 (20.5)	
Lung metastasis				0.24
Yes	16 (13.5)	4 (8.9)	12 (16.4)	
No	102 (86.5)	41 (91.1)	61 (83.6)	
Extrathoracic metastasis				0.98
Yes	105 (89.0)	40 (88.9)	65 (89.0)	
No	13 (11.0)	5 (11.1)	8 (11.0)	
Bone metastasis				0.02
Yes	35 (29.6)	6 (13.3)	29 (39.7)	
No	83 (70.4)	39 (86.7)	44 (60.3)	
Brain metastasis				0.66
Yes	34 (28.8)	14 (31.1)	20 (27.3)	
No	84 (71.2)	31 (68.9)	53 (72.7)	
Liver metastasis				0.98
Yes	34 (28.8)	13 (28.8)	21 (28.8)	
No	84 (71.2)	32 (71.2)	52 (71.2)	
Distant LN				0.72
Yes	31 (26.3)	11 (24.4)	20 (27.4)	
No	87 (73.7)	34 (75.6)	53 (72.6)	
BMI				0.36
BIM ≥ 24	60 (50.8)	21 (46.7)	39 (53.4)	
18.5 ≤ BIM < 24	56 (47.5)	24 (53.3)	32 (43.9)	
BIM < 18.5	2 (1.7)	0 (0.0)	2 (2.7)	
T Stage				0.29
T = 4	48 (40.7)	21 (46.7)	27 (37.0)	
T= 1–3	70 (59.3)	24 (53.3)	46 (63.0)	
N Stage				0.07
N = 3	70 (59.3)	22 (48.9)	48 (65.8)	
N = 0–2	48 (40.7)	23 (51.1)	25 (34.2)	
Anti-PD-L1 drugs				0.93
Atezolizumab	39 (33.0)	15 (33.3)	24 (32.9)	
Durvalumab	52 (44.0)	19 (42.2)	33 (45.2)	
Other PD-L1 antibodies	27 (23.0)	11 (24.5)	16 (21.9)	
Treatment cycles				0.17
>6 cycles	1 (0.9)	1 (2.2)	0 (0.0)	
4–6 cycles	114 (96.6)	44 (97.8)	70 (95.9)	
<4 cycles	3 (2.5)	0 (0.0)	3 (4.1)	
Response to CT-IT				
CR/PR	85 (72.0)	38 (84.4)	47 (64.4)	0.01
SD/PD	33 (28.0)	7 (15.6)	26 (35.6)	

Abbreviations: ES-SCLC: extensive-stage small-cell lung cancer; CT-IT: chemo-immunotherapy; TRT: thoracic radiation; LN: lymph node; T: tumor; N: node; BMI: body mass index; ECOG PS: Eastern Cooperative Oncology Group performance status; anti-PD-L1: anti-programmed cell death-ligand 1; CR: complete response; PR: partial response; SD: stable disease; PD: progressive disease.

**Table 2:** Univariate analysis and multivariate analysis of PFS in all patients with ES-SCLC.

Characteristics	Univariate Analysis			Multivariate Analysis		
	HR	95% CI	*p*-Value	HR	95% CI	*p*-Value
Clinicopathology						
Gender (Male vs. Female)	0.857	0.508–1.444	0.562	-	-	-
Age (>65 vs. ≤65; years)	0.700	0.458–1.071	0.100	-	-	-
Smoking (Yes vs. No)	0.882	0.522–1.488	0.637	-	-	-
Alcohol (Yes vs. No)	0.908	0.605–1.364	0.643	-	-	-
T stage (4 vs. <4)	1.065	0.716–1.583	0.757	-	-	-
N stage (3 vs. <3)	1.364	0.915–2.035	0.128	-	-	-
BMI (≥23.5 vs. <23.5)	1.441	0.968–2.145	0.072	-	-	-
Lung metastasis (Yes vs. No)	0.678	0.341–1.348	0.268	-	-	-
Extrathoracic metastasis (Yes vs. No)	1.510	0.802–2.842	0.201	-	-	-
Bone metastasis (Yes vs. No)	1.424	0.932–2.176	0.102	-	-	-
Brain metastasis (Yes vs. No)	1.429	0.927–2.204	0.106	-		-
Liver metastasis (Yes vs. No)	1.573	1.030–2.402	0.036 *	1.601	1.048–2.445	0.029 *
Distant LN (Yes vs. No)	0.831	0.528–1.306	0.422	-	-	-
TRT (Yes vs. No)	0.634	0.423–0.951	0.028 *	0.683	0.455–1.026	0.066
Response to CT-IT (CR/PR vs. SD/PD)	0.456	0.298–0.697	0.000 *	0.481	0.314–0.736	0.001 *
Hematology				-	-	-
NLR (≥1.5 vs. <1.5)	1.497	0.778–2.879	0.227	-	-	-
MLR (≥0.5 vs. <0.5)	0.893	0.556–1.435	0.640	-	-	-
PLR (≥194.4 vs. <194.4)	0.885	0.593–1.323	0.553	-		-
LDH (≥231.0 vs. <231.0)	1.225	0.823–1.821	0.317	-	-	-

Abbreviations: PFS: progression-free survival; ES-SCLC: extensive-stage small-cell lung cancer; HR: hazard ratio; CI: confidence interval; T: tumor; N: node; BMI: body mass index; LN: lymph node; NLR: neutrophils–lymphocyte ratio; MLR: monocyte–lymphocyte ratio; PLR: platelet–lymphocyte ratio; LDH: lactate dehydrogenase; TRT: thoracic radiation; *: *p* < 0.05.

**Table 3:** Univariate analysis and multivariate analysis of OS in all patients with ES-SCLC.

Characteristics	Univariate Analysis			Multivariate Analysis		
	HR	95% CI	*p*-Value	HR	95% CI	*p*-Value
Clinicopathology						
Gender (Male vs. Female)	0.814	0.425–1.557	0.533	-	-	-
Age (>65 vs. ≤65; years)	0.873	0.518–1.473	0.612	-	-	-
Smoking (Yes vs. No)	1.278	0.663–2.464	0.464	-	-	-
Alcohol (Yes vs. No)	1.266	0.761–2.106	0.363	-	-	-
T stage (4 vs. <4)	0.726	0.434–1.214	0.222	-	-	-
N stage (3 vs. <3)	2.136	1.262–3.616	0.005 *	1.281	0.723–2.267	0.396
BMI (≥31.9 vs. <31.9)	3.188	0.771–13.179	0.109	-	-	-
Lung metastasis (Yes vs. No)	1.274	0.662–2.451	0.468	-	-	-
Extrathoracic metastasis (Yes vs. No)	3.618	1.293–10.128	0.014 *	2.012	0.641–6.314	0.231
Bone metastasis (Yes vs. No)	3.101	1.874–5.132	0.000 *	2.292	1.353–3.884	0.002 *
Brain metastasis (Yes vs. No)	1.381	0.832–2.293	0.212	-		-
Liver metastasis (Yes vs. No)	3.366	2.027–5.591	0.000 *	2.889	1.710–4.879	0.000 *
Distant LN (Yes vs. No)	1.388	0.800–2.407	0.243	-	-	-
TRT (Yes vs. No)	0.522	0.305–0.893	0.018 *	0.564	0.317–1.004	0.052
Response to CT-IT (CR/PR vs. SD/PD)	0.638	0.383–1.064	0.085			
Hematology				-	-	-
NLR (≥1.6 vs. <1.6)	1.808	0.824–3.968	0.140	-	-	-
MLR (≥0.5 vs. <0.5)	0.995	0.566–1.750	0.987	-	-	-
PLR (≥175.5 vs. <175.5)	1.252	0.767–2.043	0.369	-		-
LDH (≥231.0 vs. <231.0)	1.939	1.160–3.241	0.011 *	1.421	0.833–2.422	0.197

Abbreviations: OS: overall survival; ES-SCLC: extensive-stage small-cell lung cancer; HR: hazard ratio; CI: confidence interval; T: tumor; N: node; BMI: body mass index; LN: lymph node; NLR: neutrophils–lymphocyte ratio; MLR: monocyte–lymphocyte ratio; PLR: platelet–lymphocyte ratio; LDH: lactate dehydrogenase; TRT: thoracic radiation; *: *p* < 0.05.

**Table 4:** Grade 3 and 4 adverse events in two groups of ES-SCLC patients.

Adverse Event	CT-IT + TRT Group	CT-IT Group
	(n = 45)	(n = 73)
Esophagitis	1 (2.2%)	0 (0%)
Pneumonia	2 (4.4%)	1 (1.3%)
Leukopenia	3 (6.6%)	6 (8.2%)
Neutropenia	10 (22.2%)	17 (23.2%)
Anemia	4 (8.8%)	6 (8.2%)
Thrombocytopenia	3 (6.6%)	5 (6.8%)

Abbreviations: TRT: thoracic radiotherapy; CT-IT: chemo-immunotherapy.

## Data Availability

The data supporting this study’s findings are available from the corresponding author upon reasonable request.

## Abbreviations

Thoracic radiotherapy (TRT); Extensive-stage small-cell lung cancer (ES-SCLC); Chemo-immunotherapy (CT-IT); Intensity-modulated radiotherapy (IMRT); Overall survival (OS); Progression-free survival (PFS); Adverse event (AE); Anti-programmed cell death ligand-1 (PD-L1); American Joint Committee on Cancer (AJCC); Eastern Cooperative Oncology Group (ECOG); Organs at risk (OARs); Planning target volume (PTV); Gross tumor volume (GTV); Clinical target volume (CTV); Body mass index(BMI); Tumor-node-metastasis (TNM); Response Evaluation Criteria In Solid Tumors (RECIST); Receiver operating characteristic (ROC); Lactate dehydrogenase (LDH); Lymph node (LN); Complete response (CR); Partial response (PR); Stable disease (SD); Progressive disease (PD); Prophylactic cranial irradiation (PCI); Non-small cell lung cancer (NSCLC); objective response rate (ORR).
